# Application of orthokeratology on myopia control and its effect on ocular surface and meibomian gland function in Chinese myopic adolescents

**DOI:** 10.3389/fmed.2022.979334

**Published:** 2022-12-08

**Authors:** Wang Hui, Hu Xiao-feng, Li Song-guo, Wang Jing-jing, Huang Xuan, Tao Yong

**Affiliations:** ^1^Department of Ophthalmology, Beijing Chaoyang Hospital, The Third Clinical Medical College of Capital Medical University, Beijing, China; ^2^Peking University Shougang Hospital, Beijing, China; ^3^Beijing Tongzhou Maternity and Child Health Care Hospital, Beijing, China

**Keywords:** myopia, ocular surface, meibomian gland function, orthokeratology, adolescents

## Abstract

**Purpose:**

This study aimed to investigate the influence of orthokeratology (OK) on myopia control and ocular surface and meibomian gland function in myopic adolescents.

**Methods:**

A prospective study was conducted over a 12-month period. The subjects were classified into two groups, namely, the OK lens group and the frame glasses control group. Axial length, corneal curvature, ocular surface, and meibomian gland parameters were measured at baseline, 1, 3, 6, and 12 months after wearing OK lenses.

**Results:**

The axial length growth rate in the OK group was significantly slower than in the control group (*P* < 0.01). The naked eye vision and the ocular surface disease index (OSDI) scores recorded 1, 3, 6, and 12 months after wearing OK lenses were significantly higher than the scores recorded before wearing OK lenses. There was no significant difference in other ocular parameters at each follow-up time point compared with pre-wearing (*P* > 0.05). After using the OK lens for 6 months, the OSDI score and corneal fluorescein staining (CFS) score increased significantly (*P* < 0.001), but there were no significant differences in other parameters among the groups. No infectious keratitis occurred during the study.

**Conclusion:**

These results provide evidence that the use of OK lenses can control the axial growth and progress rate of myopia compared with frame glasses. During the 12-month follow-up, although wearing OK lenses may have aggravated dry eye symptoms, each patient’s ocular surface and meibomian gland function did not change significantly, indicating that the use of OK lenses is a relatively safe modality for the control of myopia in adolescents.

## Introduction

Myopia can cause visual impairment and its prevalence rates have been increasing worldwide (1). High myopia is a major cause of blindness due to its many associated complications, such as retinal detachment and macular choroidal degeneration, which have a great impact on visual function (2–5). The incidence of myopia among Chinese adolescents has increased significantly. Multiple clinical studies have shown that the use of overnight orthokeratology (OK) lenses is an effective method for slowing down myopic progression in adolescents (6–10).

With the rapid increase in using OK lenses worldwide, potential complications have become a significant concern among parents. Long-term direct contact with the cornea results in a certain influence on the corneal layer (11). Potential complications include keratitis and corneal staining. Keratitis continues to be the most serious complication associated with OK. In earlier reports of keratitis in Chinese patients, the attributable factors included a lack of both practitioner training and routine follow-ups (12, 13). In addition, corneal staining has commonly been reported in patients wearing OK lenses, and overnight OK lenses have also been associated with reduced basal tear secretion and tear film stability (14, 15).

However, limited studies have assessed the effect of OK lenses on the corneal epithelium and ocular surface function among Chinese adolescents. Given that many Chinese children wear OK lenses for myopia control, it is crucial to elucidate the long-term safety of wearing OK lenses. This study was conducted to investigate the influence of OK lenses on myopia control and on the ocular surface and meibomian gland function.

## Materials and methods

### Subjects

This was a prospective study conducted at Beijing Chaoyang Hospital, the Third Clinical Medical College of Capital Medical University; Peking University Shougang Hospital; and Beijing Tongzhou Maternity and Child Health Hospital, China from January 2021 to December 2021. OK lens wear in 45 eyes and frame glasses wear in 40 eyes with myopia up to −5.5 D were included in the study. Clinical data, including sex, age, refraction, visual acuity, prescription lens power, adverse events (AEs), and complications were recorded. All procedures in this study were performed in accordance with the standards of the Declaration of Helsinki 2013 and were approved by the Institutional Review Board of Beijing Chaoyang Hospital. Written informed consent was obtained from all patients and their parents for being included in the study.

Inclusion criteria include patients in the age bracket of 8–15 years; with myopia up to –5.0 D; and with-the-rule astigmatism of up to –1.5 D or against-the-rule astigmatism up to –0.75 D with keratometry values between 40.0 and 45.0 D. Exclusion criteria include patients with pathological myopia, corneal pathologies, ocular surgery, keratoconus, and systemic comorbidities.

Patients were classified into two groups, namely, the study group (OK group) was prescribed OK lenses, and the control group was prescribed daily wear frame glasses in both eyes. All OK patients were fitted with reverse geometry OK lenses (α ORTHO-K; ALPHA Corp., Nagoya, Japan). The lens material used for all patients was Boston EM (Polymer Technology Corporation, Rochester, NY, USA). Adolescent patients were recommended to wear OK lenses for at least 8 consecutive hours every day.

### Ophthalmic examination

All the patients and controls had ophthalmic examinations. Naked eye vision, best-corrected visual acuity (BCVA), corneal topography, corneal endothelial cell count (CECC), spherical equivalent (SE), intraocular pressure (IOP) and axial length (AL; IOL-Master, Carl Zeiss Meditec AG, Jena, Germany) analyses were evaluated at every visit. Follow-up was performed on 1st day, 1 month, 3 months, 6 months, and 12 months after the start of treatment. At 12 months after wearing OK lens, the AEs were recorded. Ocular examinations of each participant were carefully assessed by senior pediatric ophthalmologists.

The ocular surface and meibomian gland examinations were also evaluated during the follow-up period.

### Questionnaire and anterior segment assessment dry eye

The ocular surface disease index (OSDI) questionnaire was used for evaluating the dry eye severity in all patients. A corneal fluorescein staining (CFS) score and a tear break-up time (TBUT) were carried out using a slit lamp. The grading of CFS was as follows: Grade 0: a few dots of staining; Grade 1: increased spots showing a scatter pattern; Grade 2: obvious clinical manifestations and dense and diffuse spots; Grade 3: many stippling stains, aggregation, and fusion occurred in a large range; and Grade 4: diffuse stippling stains of the whole cornea, mass fusion, or even complete epithelial loss. The Keratograph^®^ 5M software was used to obtain the tear film stability information automatically.

**FIGURE 1:**
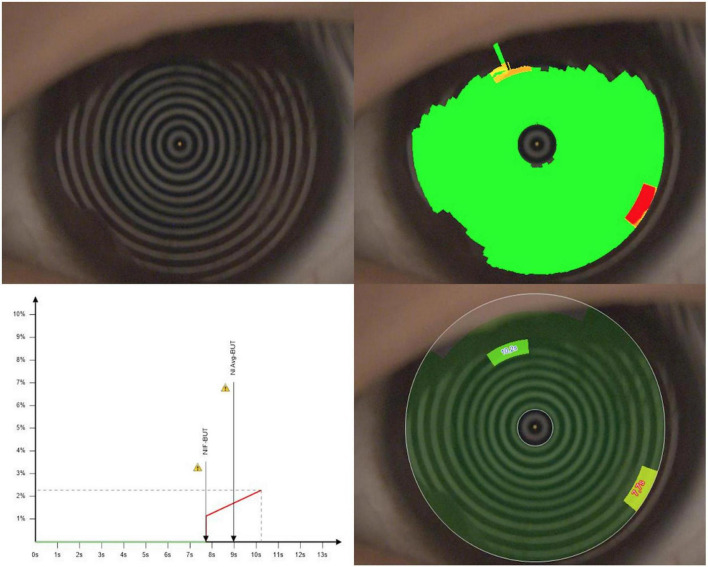
The first tear break-up time (f-TBUT) and average tear break-up time (a-TBUT) measured with the Keratograph 5M software.

**FIGURE 2:**
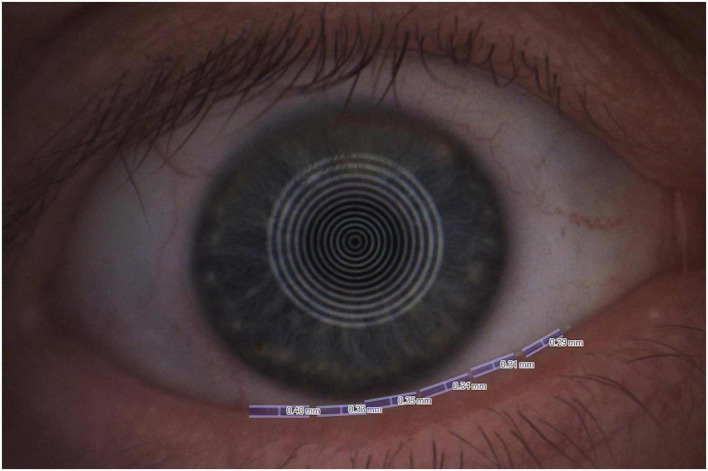
The non-invasive tear meniscus height (TMH) measured with the Keratograph 5M software.

Dynamic tear film stability assessment. The following four indices were selected to analyze the ocular surface function: (1) first tear break-up time (f-TBUT); (2) average tear break-up time (a-TBUT); (3) number of final break-up areas (Figure 1); and (4) tear meniscus height (TMH) (Figure 2) (16).

### Meibomian gland examination

**FIGURE 3:**
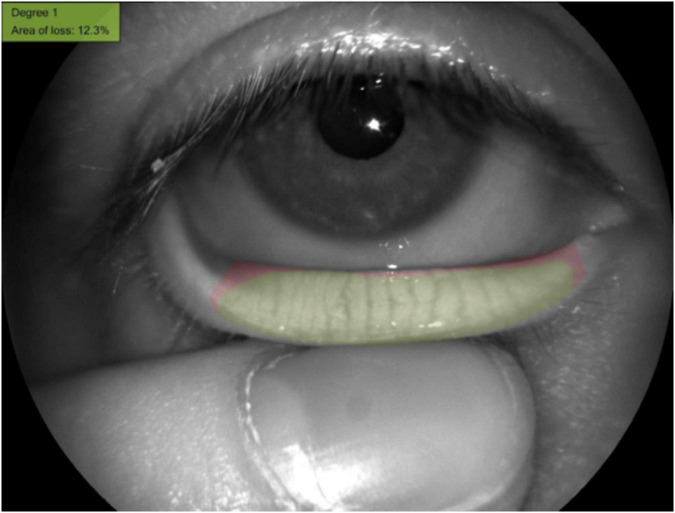
The meibomian gland loss area measured with the Keratograph 5M software.

The meibomian gland loss area of the lower eyelid was evaluated by the instrument of Keratograph 5M (Figure 3). The degree of meibomian gland loss area is as follows: Grade 0: no loss of meibomian gland; Grade 1: loss of <1/3 of the whole gland area; Grade 2: loss of 1/3∼2/3 of the whole gland area; and Grade 3: loss of >2/3 of the whole gland area.

### Statistical analysis

The Kolmogorov–Smirnov test was used to identify the normality of distribution. Descriptive statistics were calculated as the mean and standard deviation for normally distributed variables. An independent sample *t*-test for normal distributions and the Kruskal–Wallis tests were used to compare the continuous variables between the OK and control groups, whereas a paired *t*-test was used to compare the change of measurement results for paired samples. The differences in the baseline ocular parameters and subsequent changes during the follow-up period were analyzed using the Kruskal–Wallis test and the *post-hoc* tests with Bonferroni correction. A *p*-value of <0.05 was regarded as statistically significant. All reported *P*-values were two-sided. Statistical analysis was performed using the SPSS software version 26 (SPSS, Inc., IL, USA).

## Results

### Demographic data and clinical characteristics

The demographic data and clinical characteristics are shown in Table 1. The mean SE in the OK lenses group was −2.27 ± 1.08 D and −1.88 ± 0.99 in the control group (*P* = 0.347). The IOP was 15.31 ± 1.66 mmHg in the OK group and 15.45 ± 1.40 mmHg in the control group (*P* = 0.113). There was no difference in the naked eye vision, AL, corneal apical power, corneal equivalent power, corneal keratometry astigmatism, and CECC as well as their difference values between the two groups (all *P*-values > 0.05) (Table 2).

**TABLE 1:** Demographic data and clinical characteristics of the patients.

	OK group	Control group	*P*-value
Number of eyes (*n*)	45	40	–
SE (D), mean ± SD	−2.27 ± 1.08	−1.88 ± 0.99	0.347^†^
Naked eye vision, mean ± SD	0.74 ± 0.44	0.72 ± 0.38	0.795^†^
IOP (mmHg), mean ± SD	15.31 ± 1.66	15.45 ± 1.40	0.113^†^
AL (mm), mean ± SD	24.09 ± 0.59	24.06 ± 0.63	0.616^†^
Corneal apical power (D), mean ± SD	43.51 ± 1.05	43.61 ± 1.08	0.808^†^
Corneal equivalent power (D), mean ± SD	43.25 ± 1.04	43.25 ± 1.00	0.863^†^
Corneal keratometry astigmatism (D), mean ± SD	0.53 ± 0.31	0.73 ± 0.40	0.117^†^
CECC, mean ± SD	2688.87 ± 285.38	2608.83 ± 258.38	0.550^†^

AL, axial length; CECC, corneal endothelial cell count; D, diopter; IOP, intraocular pressure; OK, orthokeratology; SE, spherical equivalent; SD, standard deviation.

^†^Independent sample *t*-test.

**TABLE 2:** Ophthalmology examination between the orthokeratology (OK) group and control group after 12 months.

	OK group (12 months)	Control group (12 months)	*P*-value
SE (D), mean ± SD	40.97 ± 0.87	43.24 ± 0.99	0.980^†^
AL (mm), mean ± SD	24.21 ± 0.57	24.40 ± 0.61	0.148^†^
AL growth (mm), mean ± SD	0.07 ± 0.01	0.21 ± 0.03	<**0.001**^†^*
Corneal apical power (D), mean ± SD	41.32 ± 0.96	43.58 ± 1.10	0.466^†^
Corneal apical power change (D), mean ± SD	2.20 ± 0.60	0.03 ± 0.28	<**0.001**^†^*
Corneal equivalent power (D), mean ± SD	41.64 ± 0.87	43.19 ± 1.02	0.472^†^
Corneal equivalent change (D), mean ± SD	2.28 ± 1.04	0.73 ± 0.40	<**0.001**^†^*
Corneal keratometry astigmatism (D), mean ± SD	0.69 ± 0.58	0.67 ± 0.48	0.280^†^
CECC, mean ± SD	2807.49 ± 95.85	2780.68 ± 95.94	0.852^†^
IOP (mmHg), mean ± SD	15.13 ± 1.55	14.60 ± 1.55	0.955^†^
Naked eye vision, mean ± SD	−0.02 ± 0.06	0.79 ± 0.35	<**0.001**^†^*
TBUT (s), mean ± SD	11.97 ± 1.08	12.00 ± 0.82	0.914^†^

AL, axial length; CECC, corneal endothelial cell count; D, diopter; IOP, intraocular pressure; OK, orthokeratology; SE, spherical equivalent; TBUT, tear break-up time, SD, standard deviation.

^†^Independent sample *t*-test.

*Statistically significant.

Bold values indicate *P* < 0.05.

### Ophthalmology examination between orthokeratology group and control group after 12 months

Axial length growth in the OK group (0.07 ± 0.01 mm) was slower than that found in the control group (0.21 ± 0.03 mm), with a statistically significant difference between groups (*P* < 0.01). The corneal apical power change and corneal equivalent change of patients in the OK group were significantly higher than that in the control group (all *P*-values < 0.05). Naked eye vision in the OK and control groups at 12 months was –0.02 ± 0.06 and 0.79 ± 0.35 log MAR, respectively (*P* < 0.05). There was no difference in SE, AL, corneal apical power, corneal equivalent power, corneal keratometry astigmatism, CECC, IOP, and TBUT (all *P*-values > 0.05).

### The baseline parameters and changes during the follow-up period

Compared with the baseline values, the naked eye vision at 1, 3, 6, and 12 months after wearing OK lenses was significantly increased in comparison with the pre-wearing level (all *P*-values < 0.05). There was no significant difference in other ocular parameters (AL, CECC, and IOP) at each follow-up time point compared with before wearing OK lenses level (all *P*-values > 0.05).

The OSDI scores increased with the wearing of OK lenses during a follow-up period (*P* < 0.05, for 1, 3, 6, and 12 months vs. baseline). The CFS score at 1 month after wearing OK lenses significantly increased in comparison with before wearing OK lenses level (*P* < 0.05). However, there were no significant differences in the other parameters (f-TBUT, a-TBUT, TMH, final break-up areas, and meibomian gland loss grade) during a follow-up period (all *P*-values > 0.05) (Table 3).

**TABLE 3:** The baseline ocular parameters and subsequent changes during the follow-up period.

	Pre lens wear	1 month	3 months	6 months	12 months
SE (D), mean ± SD	−2.27 ± 1.08	–	–	–	–
AL (mm), mean ± SD	24.09 ± 0.59	24.12 ± 0.59	24.16 ± 0.60	24.23 ± 0.61	24.22 ± 0.57
Corneal curvature (D), mean ± SD	43.25 ± 1.04				
CCT (μ m), mean ± SD	553.71 ± 9.83	550.38 ± 9.72	554.04 ± 12.87	555.71 ± 10.15	555.69 ± 9.42
CECC, mean ± SD	2726.64 ± 270.45	2743.69 ± 28.47	2787.84 ± 101.31	2751.89 ± 72.38	2794.15 ± 87.02
IOP (mmHg), mean ± SD	15.31 ± 1.66	15.18 ± 1.51	15.00 ± 1.68	14.96 ± 2.01	15.13 ± 1.55
Naked eye vision, mean ± SD	0.74 ± 0.44	−0.01 ± 0.02	−0.02 ± 0.04	−0.02 ± 0.07	−0.03 ± 0.09
OSDI score, mean ± SD	5.36 ± 1.15	6.36 ± 2.52	7.26 ± 1.72	7.16 ± 2.09	7.42 ± 1.40
TBUT (s), mean ± SD	11.98 ± 1.08	11.87 ± 0.97	11.80 ± 1.04	12.00 ± 1.00	11.87 ± 1.16
CFS score, mean ± SD	0.24 ± 0.43	0.42 ± 0.54	0.40 ± 0.54	0.36 ± 0.48	0.38 ± 0.61
f-TBUT (s), mean ± SD	11.85 ± 0.84	11.93 ± 1.06	12.24 ± 0.79	11.92 ± 1.08	11.90 ± 1.45
a-TBUT (s), mean ± SD	13.45 ± 1.25	13.38 ± 1.19	13.56 ± 1.08	13.21 ± 1.23	13.18 ± 1.00
TMH (mm), mean ± SD	0.25 ± 0.06	0.24 ± 0.06	0.23 ± 0.05	0.23 ± 0.04	0.24 ± 0.06
Final break-up areas, mean ± SD	3.67 ± 1.98	4.12 ± 2.08	3.99 ± 2.23	4.11 ± 1.88	4.31 ± 2.24
Meibomian gland loss grade, mean ± SD	0.44 ± 0.50	0.42 ± 0.50	0.44 ± 0.50	0.42 ± 0.50	0.47 ± 0.55

AL, axial length; CCT, central corneal thickness; CFS, corneal fluorescein staining; D, diopter; IOP, intraocular pressure; OK, orthokeratology; OSDI, ocular surface disease index; SE, spherical equivalent; TBUT, tear break-up time; TMH, tear meniscus height.

### Ocular surface presentations before and after wearing the orthokeratology lens

It was found that after using OK lenses for 12 months, the OSDI score and CFS score increased significantly in comparison with the pre-wearing level (all *P*-values < 0.001). There were no significant differences in other parameters (TBUT, f-TBUT, a-TBUT, and TMH) among the groups (Table 4).

**TABLE 4:** The ocular surface parameters and subsequent changes after wearing the OK lens.

	Pre-lens wear	Post-lens use (12 months)	*P*-value
OSDI score, mean ± SD	5.36 ± 1.15	7.42 ± 1.40	<**0.001**^†^*
TBUT (s), mean ± SD	11.98 ± 1.08	11.87 ± 1.16	0.506^†^
CFS score, mean ± SD	0.24 ± 0.43	0.38 ± 0.61	**0.010**^†^*
f-TBUT(s), mean ± SD	11.85 ± 0.84	11.90 ± 1.45	0.566^†^
a-TBUT (s), mean ± SD	13.45 ± 1.25	13.18 ± 1.00	0.472^†^
TMH (mm), mean ± SD	0.25 ± 0.06	0.24 ± 0.06	0.655^†^
Final break-up areas, mean ± SD	3.67 ± 1.98	4.31 ± 2.24	0.499^†^
IOP (mmHg), mean ± SD	15.31 ± 1.67	15.13 ± 1.55	0.408^†^
AL (mm), mean ± SD	24.09 ± 0.59	24.22 ± 0.57	0.887^†^

AL, axial length; CFS, corneal fluorescein staining; IOP, intraocular pressure; OK, orthokeratology; OSDI, ocular surface disease index; TBUT, tear break-up time; TMH, tear meniscus height.

^†^Independent sample *t*-test.

*Statistically significant.

Bold values indicate *P* < 0.05.

### Adverse events

The AEs during the 12-month follow-up period are shown in Table 2. In the OK group, a total of 12 AEs were observed in 45 eyes; no AEs were found in the remaining 29 eyes. Allergic conjunctivitis and cornea epithelial keratopathy were the most frequent AEs. Infectious keratitis was not found throughout the study period.

## Discussion

Myopia is becoming a major public health problem worldwide. Holden et al. (17) have estimated the prevalence of myopia in the South Asia region to be around 20% in 2010, 38% in 2030, and 53% in 2050. Over the past decades, different strategies have been proposed for the prevention or control of myopic progression, including atropine eye drops, increased outdoor activity, and use of OK lenses. The use of OK lenses is an effective means of myopia control, but the possible side effects have raised considerable concerns.

Numerous studies have confirmed the effect of OK in controlling myopic progression in schoolchildren (6–8, 18). The OK lens corrects myopia through its reverse geometrical design: the basic arc is flatter than the center corneal part, whereas the position of the reverse arc is steeper. As the patients wear the lens, mechanical pressure is combined with a massaging effect through palpebral activities on the lens to reshape the cornea. Then, the central part of the cornea becomes thinner and flatter, while the mid-peripheral part becomes thicker and steeper. The corneal central curvature is, therefore, reduced, and myopia is corrected. OK lenses work by changing the shape of the cornea and reducing the central corneal curvature (19). The use of OK lenses was reported to be effective in slowing the progression of myopia with a clinically acceptable safety profile (20). Two studies have evaluated the long-term efficacy of wearing OK lenses in terms of reducing the axial elongation rate and controlling myopic progression (21, 22). Hiraoka et al. (23) believed the long-term efficacy and safety of OK lens wear in reducing myopic progression. In this study, the diopter was reduced after the first use of overnight OK lenses wear. The OK lens-wearing eyes showed significantly less axial elongation compared to the control eyes. There has been much speculation about the possible mechanisms of OK in myopia control. The OK lenses work by altering the corneal shape from prolate to oblate, resulting in a reduction in the central corneal curvature. This alteration may cause a redistribution of corneal epithelium and anterior stromal tissue over the central treatment zone which is 5–6 mm in diameter (24).

Previous studies have confirmed that hyperopic defocus on the peripheral retina induces axial eye growth and the development of myopia, while myopic defocus induces a decrease in the eye growth rate in animal models (25, 26). Hyperopic defocus often occurs when myopic patients wear traditional frame glasses (27). Corneal reshaping after wearing OK lenses has been shown to induce myopic defocus (28), and this may be the mechanism underlying the myopia control effects. Swarbrick et al. (10) found that the short-term axial shortening may represent choroidal thickening due to a reduction or neutralization of the myopiogenic stimulus of eye growth, which may result from wearing OK lenses. They also speculated that any choroidal thickening would move the retinal pigment epithelium forward, giving rise to an apparent shortening of the eye (10).

The use of OK lenses is an effective means of myopia control; however, there are concerns about its possible side effects as it is mainly used on children and teenagers. The most frequent problems are dry eye and ocular surface discomfort, caused by tear film instability (29). Wang et al. (29) reported an increase in OSDI scores after their patients wore OK lenses. In the present study, OSDI scores in the OK group improved after wearing OK lenses. Wearing OK lenses can lead to the tear film instability, resulting in ocular surface discomfort, which is also considered a risk in children wearing OK lenses (29–31). Wearing OK lenses can reduce basal tear secretion and tear film stability (15). The subsequent ocular surface discomfort that results from tear film instability is also considered a risk in children wearing OK lenses. The possible mechanisms responsible for inducing tear film instability include thinning of the tear film lipid layer and conjunctival metaplasia (29, 32–34). Na et al. (31) found that meibomian gland dysfunction occurred after wearing the OK lens for 1 year, which affected the evaporation rate of the tear and caused a decrease in tear film stability. Wang et al. (29) reported that TMH was significantly increased at 1 and 3 months compared with the pre-wearing level but did not affect the meibomian gland function. In the study reported by Cho, there was no significant difference in TMH and TBUT between children with different myopia treatment methods and normal children (35). Xie et al. (30) showed that the use of OK lenses did not affect basic tear secretion and ocular surface inflammation in myopic children. In the present study, TMH and TBUT after using OK lenses were not significantly different from pre-lens wear.

Corneal staining is another common condition reported in patients wearing OK lenses. Chan et al. reported in their study that corneal staining was the most common complication associated with the use of DreimLens and eLens ortho-K lenses (composed of Boston XO), and after the first overnight use of the lenses, corneal staining was observed in 41% of patients (36). Li et al. (14) found that corneal epithelial staining increased and the stability of tears decreased after wearing OK lenses (composed of Boston XO). In the present study, the CFS score recorded after wearing OK lenses (composed of Boston EM) was significantly higher than the score before wearing OK lenses. It is worth noting that there is variation in the oxygen permeability coefficient of the different materials used in OK lenses and that the oxygen permeability of the OK lenses worn in our study was better than that of the lenses worn in previous studies. However, all the tested OK lenses cause corneal staining during wearing. Chan et al. (36) suggested that corneal staining was caused by mechanical abrasion of the cornea after wearing the OK lens. Xie et al. (37) pointed out that peripheral punctate staining after using OK lenses was more commonly associated with preexisting conditions such as misdirected lashes. Many parents are also concerned about the long-term effects of wearing OK lenses on the corneal endothelium. No significant changes in endothelial cell density or corneal polymegethism have been reported to date, indicating that the overnight OK lens does not negatively affect endothelial cells. The results of this study align with those of previous studies: the CECC and the CCT did not change significantly.

Although OK has a relatively low rate of AEs, they are still a cause for concern, and efforts should be made to reduce their occurrence. Infectious keratitis remains the most concerning complication (12, 38–40). Since 2001, there has been a steady stream of case series and case reports of microbial keratitis associated with the overnight OK lens. Bullimore et al. (41) summarized the literature and reported that most cases of microbial keratitis in OK were reported from East Asia. The main reasons were that overnight OK lens wear may reduce the ocular surface’s defense against infection (42). Sporadic keratitis was reported in earlier Chinese publications, and the attributable factors included a lack of training of practitioners and a lack of routine follow-ups (12, 43). The OK lenses may disrupt the corneal epithelium and extended overnight wear may potentiate infectious keratitis. Kam et al. reported that *Pseudomonas aeruginosa* and *Acanthamoeba* are the most identified infectious agents in infectious keratitis associated with the use of OK lenses (40), which deserves special attention from ophthalmologists. Wearing OK lenses may also make patients more prone to allergic conjunctivitis due to two possible reasons. One is that the patient has pre-existing but previously undetected symptoms of allergic conjunctivitis. The other is that wearing OK lenses may worsen the symptoms of allergic conjunctivitis. Na et al. reported that wearing OK lenses aggravated existing allergic conjunctivitis in patients with a history of allergic conjunctivitis (31). Arita et al. (44) reported that perennial allergic conjunctivitis is associated with increased meibomian gland duct distortion. The incidence of allergic conjunctivitis in our subjects at the 12-month follow-up was 15.6%. Of these seven subjects, three had a history of allergic conjunctivitis before enrolling in this study, and four only had allergic conjunctivitis symptoms for 12 months after wearing OK lenses.

The risk factors associated with AE incidence in OK wearers had also been discussed earlier. It is reported that younger age, a higher degree of myopia, and allergic conjunctivitis were associated with higher corneal AE incidence in OK wearers, and a higher degree of myopia was a risk factor for significant AE (45). Lipson et al. (46) reported that younger children showed a trend toward more corneal staining than older children. A higher degree of myopia was a significant risk factor for corneal AE after wearing the OK lens (46, 47). Hu et al. (45) reported that there was a higher association between AC and corneal AE incidence with an odds ratio (OR) of 1.706. The ocular inflammation, tear film instability, and potential mechanical injury due to eye-rubbing were assumed to play significant roles in causing the corneal AEs.

The present study has some potential limitations. First, some of the data, such as the OSDI and tear film stability parameters, were subjective. Second, the reasons for the increased risk of conjunctivitis in patients wearing OK lenses should be analyzed. In this study, only the ocular surface and the meibomian gland function were analyzed but not the inflammatory factors of tears. Third, choroidal thickness was not measured in this study, so a causal relationship between wearing OK lenses and choroidal thickness changes cannot be speculated.

The results of this study also raise important questions that can be addressed in future research, namely: Does wearing OK lenses cause the upregulation of tear inflammatory factors, thereby increasing the probability of OK lens-wearers developing conjunctivitis? What is the mechanism for inhibition of axial length growth in overnight OK lenses? Is there a relationship between structural changes in the choroid and axial length shortening or delayed elongation? Hence, further studies are warranted to clarify the speculations arising from this study.

## Conclusion

The findings of this study have provided further evidence of the control effect of OK lenses; OK can effectively control the progression of myopia and axial length elongation. During the 6-month follow-up, the patients’ cornea, ocular surface, and meibomian gland function did not change significantly, indicating that the use of OK lenses is a safe modality for the correction of myopia in children and adolescents. Although allergic conjunctivitis and hordeolum developed in individual patients during follow-up, it did not affect the patient’s cornea or ocular surface. The tip is that the tear dynamics and the meibomian gland structure should be carefully monitored when OK lenses are being used. Given that OK has been shown to delay the progression of myopia (a critical issue in myopia prevention) and not to affect the ocular surface, it is a potential option for clinicians to consider as part of the myopia prevention process.

## Data availability statement

The raw data supporting the conclusions of this article will be made available by the authors, without undue reservation.

## Ethics statement

The studies involving human participants were reviewed and approved by the Institutional Review Board of Beijing Chaoyang Hospital. Written informed consent to participate in this study was provided by the participants’ legal guardian/next of kin. Written informed consent was obtained from the minor(s)’ legal guardian/next of kin for the publication of any potentially identifiable images or data included in this article.

## Author contributions

WH, HX, and TY involved in the design of the study. WH and HX conducted the study. WJ-J contributed to the collection, patients epidemiological survey, and baseline data statistics. LS-G contributed to the patient follow-up data collection. WH prepared the manuscript. WH and HX-F contributed to the critical revision of the manuscript. All authors reviewed and approved the final manuscript.
